# Association between neutrophils and renal impairment of rheumatoid arthritis: A retrospective cross‐sectional study

**DOI:** 10.1002/iid3.459

**Published:** 2021-05-25

**Authors:** Pei‐Dan Yang, Kai‐Jun Zhu, Si‐Min Lin, Zhi‐Xin Chen, Min‐Ying Liu, Qing‐Ping Liu, Chang‐Song Lin, Cong‐Qiu Chu, Qiang Xu

**Affiliations:** ^1^ Department of Rheumatology The First Affiliated Hospital of Guangzhou University of Chinese Medicine Guangzhou China; ^2^ The First Medical School Guangzhou University of Chinese Medicine Guangzhou China; ^3^ Department of Rheumatology Zhengzhou Second Hospital Zhengzhou China; ^4^ Chinese Medicine Department South China Agricultural University Hospital Guangzhou China; ^5^ Division of Rheumatology Oregon Health & Science University Portland Oregon USA

**Keywords:** cross‐sectional study, neutrophils, renal impairment, rheumatoid arthritis

## Abstract

**Objective:**

Previous studies have shown that increased neutrophils, as a manifestation of oxidative stress, may be involved in the progression of kidney disease. To our knowledge, little is known about the relationship between neutrophils and renal impairment in rheumatoid arthritis (RA). Therefore, we aim to investigate whether neutrophil is associated with renal impairment in RA patients.

**Methods:**

We retrospectively investigated the renal function of 602 RA patients in the First Affiliated Hospital of Guangzhou University of Traditional Chinese Medicine by estimated glomerular filtration rate (eGFR) from September 2018 and September 2019. The exposure variable was neutrophils, and the main outcome was eGFR. General data (gender, age, duration, hypertension, diabetes, hobbies, and medication history), whole blood markers, lipid indexes, and inflammatory indexes were collected as much as possible. We used multivariable logistic regression analysis to evaluate the association between neutrophils and renal impairment in RA participants.

**Results:**

A total of 89 cases (14.8%) had renal impairment with eGFR < 60 ml/min/1.73 m^2^, and 75 cases (84.3%) were female. Subgroup analysis showed that female (odds ratio [OR] = 0.523, 95% confidence interval [CI]: 0.318–0.867, *p* = .011), neutrophils greater thsn 7.5 × 10^9^/L (OR = 2.314, 95% CI: 1.310–4.087, *p* = .004), NLR > 3.53 (OR = 1.757, 95% CI: 1.104–2.799, *p* = .018), hemoglobin less than 120 g/L (OR = 2.413, 95% CI: 1.418–4.118, *p* = .001), and UA > 360 μmol/L (OR = 6.052, 95% CI: 3.708–9.878, *p* < .001) was related to renal damage in RA. Adjusted for several confounders, the multivariable analysis indicated that neutrophils greater than 7.5 × 10^9^/L (OR = 1.784, 95% CI: 1.164–3.288, *p* = .031) was independently associated with an increased risk of renal impairment in RA.

**Conclusion:**

Our study demonstrated that neutrophils greater than 7.5 × 10^9^/L was associated with a high risk of renal impairment in RA, suggesting that neutrophil may be a biomarker for renal impairment in RA.

## Graphical abstract

In this present study, neutrophil greater than 7.5 × 10^9^/L may be associated with a high risk of renal impairment in RA. We also found that UA > 360 μmol/L and hemoglobin less than 120 g/L independently associated with a higher risk of renal impairment in RA patients. Although more prospective studies are needed to verify our results, based on the above data, we believe that continuous monitoring of these risk factors in RA patients is necessary and it could be used for early detection and identification of changes in GFR, allowing doctors to treat patients with potential kidney damage promptly.

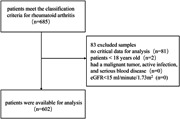

## 1 INTRODUCTION

Renal impairment, defined by an estimated glomerular filtration rate (eGFR) < 60 ml/min/1.73 m^2^ for at least 3 months,^1^ was more likely to occur in patients with rheumatoid arthritis (RA).^2^ Previous studies showed that the prevalence of renal damage in RA patients was between 5% and 50%^3^, ^4^ in different cohorts. Severe renal impairment inevitably brought physical injury and economic burden as well as death.^5^, ^6^ It was reported that the mortality rate in RA patients with kidney damage was significantly increased when compared to patients with normal renal function.^7^, ^8^ The leading causes of death amongst RA patients with renal impairment were chronic infection^3^, ^9^，inflammatory response,^10^, ^11^ severe drug adverse reactions (cyclosporin A, etanercept, COX‐2 inhibitor, NSAIDs), and vasculitis.^12^ A meta‐analysis revealed that a significantly increased risk of kidney damage in patients with RA, with a pooled risk ratio of 1.52, and a 95% confidence interval (CI) of 1.28–1.80.^13^ Consequently, it is essential to learn more about possible risk factors for renal insufficiency in patients with RA. It was well known that uric acid (UA) levels,^4^ inflammation‐related indicators, such as erythrocyte sedimentation rate (ESR),^2^ and cardiovascular (CV) risk factors,^3^, ^14^, ^15^ such as hypertension, dyslipidemia, diabetes, and obesity were related to kidney damage. Inflammation could promote the progression of RA with renal damage, and the increase of neutrophils was an indicator of inflammation. However, whether high neutrophils increase the risk of kidney damage in RA patients is currently unclear. Therefore, we conducted a cross‐sectional study to determine a potential relationship between neutrophils and renal impairment in RA patients.

## 2 METHODS

### 2.1 Study design and patients

**Figure 1:**
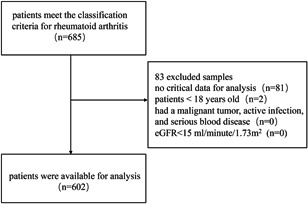
Data inclusion process

A total of 685 data from the original database of the First Affiliated Hospital of Guangzhou University of Chinese Medicine between September 2018 and September 2019, were screened in our cross‐sectional analysis. Patients in this data set should meet the classification criteria of the American College of Rheumatology for RA 2010^16^ and were over 18 years of age. Exclusion criteria were as follows: (1) no critical data for analysis; (2) participant less than 18 years old; (3) patient had a malignant tumor, active infection, and serious blood disease; (4) eGFR < 15 ml/min/1.73 m^2^. As a result, 602 samples were available for investigation. The inclusion process was shown in Figure 1.

### 2.2 Ethics statement

Written informed consent was acquired from all participants. We followed the principles of the Declaration of Helsinki and received approval from the Ethics Committee of the First Affiliated Hospital of Guangzhou University of Traditional Chinese Medicine (No. ZYYECK [2019]040).

### 2.3 Demographic and laboratory data

General demographics included sex, age (years), durations, current alcohol, cigarette use, and medication history (steroids, bDMARDs, and NSAIDs). Laboratory indicators, such as neutrophil, neutrophil to lymphocyte ratio (NLR), white blood cell (WBC), lymphocyte, red blood cells (RBC), hemoglobin levels, total cholesterol (TC), triglycerides (TG), high‐density lipoproteins (HDL), low‐density lipoproteins (LDL), ESR, C‐reactive protein levels (CRP), UA levels, rheumatoid factor, and anti‐cyclic citrullinated peptide.

### 2.4 Assessment of kidney function

The main outcome was eGFR. Following the 2013 Kidney Disease: Improving Global Outcomes (KDIGO) guidelines, the Chronic Kidney Disease—Epidemiology Collaboration (CKD‐EPI) equation involving SCr and CysC levels was used to calculate eGFR. Patients with eGFR<60 ml/min1.73m^2^ were identified as having renal impairment regardless of kidney markers.

### 2.5 Statistical analysis

The normality of the data was evaluated by the Shapiro–Wilk test.^17^ Quantitative data were presented as mean ± *SD* or median (Interquartile range [IQR]), and qualitative data as number and percentage. Categorical data were assessed by the *χ* ^2^ test or Fisher exact test, while continuous data were appraised by the Mann–Whitney test or Student *t* test. We performed subgroup analyses of eGFR on RA participants including age, gender, duration, hypertension, diabetes, current drinking, current smoking, steroid, bDMARDs, NSAIDs, neutrophils, NLR, hemoglobin, TC, LDL, HDL, TG, ESR, CRP, and UA. To further analyzed factors independently associated with the RA patients with renal impairment, we employed multivariable logistic regression analysis by establishing two models: unadjusted, adjusted I (covariates: age, duration, CRP, ESR, TG). Features with significant significance and clinical relevance in subgroup analysis were introduced into the multivariate logistic model. Results were presented as the odds ratio (OR) and their 95% confidence interval (95% CI). All analyses in our study used Stata (version 15.0) and the threshold for statistical significance was set to 0.05.

## 3 RESULTS

### 3.1 Demographic and clinical characteristics

Table 1 showed detailed information on the demographic and clinical characteristics of the RA patient cohort. Of 602 RA patients, 89 (14.8%) had renal damage with eGFR < 60 ml/min/1.73 m^2^, and 513 (85.2%) had normal renal function with eGFR > 60 ml/min/1.73 m^2^. Among renal damage group, 75 (84.3%) were female, and 31 (34.8%) suffered from Hypertension and 12(13.5%) had diabetes mellitus. Compared to RA patients with normal renal function, RA patients in the renal impairment group had a relatively older age (median [IQR]: 61 [54–68.5] vs. 59 [50–65.5], *p* = .027), lower RBC count (median [IQR]: 3.70 [3.36–4.25] vs. 3.99 [3.70–4.31], *p* = .001) and hemoglobin level (median [IQR]: 103 [87.5–116.5] vs. 110 [98–121], *p* = .006). WBC count (median [IQR]: 7.36 [5.74–10.06] vs. 6.84 [5.43–8.51], *p* = .035], TG levels (median [IQR]: 1.11 [0.91–1.41] vs. 0.97 [0.74–1.32], *p* = .003), ESR (median [IQR]: 55 [26.5–76.5] vs. 42 [22.5–63.0], *p* = .019), CRP (median [IQR]: 36.9 (15.35–76.8] vs. 23.3 [19.22–62.40], *p* = .016), UA levels (median [IQR]: 358 [269.5–441.5] vs. 270 [214–324.5], *p* < .001), Scr (median [IQR]: 88 [77.5–103] vs. 59 [50–68], *p* < .001), and CysC (median [IQR]: 1.50 [1.33–1.75] vs. 0.97 [0.85–1.11], *p* < .001) significantly increased compared with that of RA participants with normal renal function.

**Table 1:** Baseline characteristics of RA participants according to glomerular filtration rate

	**With RI**	**Without RI**	***p***
	**eGFR < 60**	**eGFR ≥ 60**	
	**(*n* = 89)**	**(*n* = 513)**	
Age (years)	61 (54‐68.5)	59 (50–65.5)	.027
Sex (%)			.206
Male	14 (15.7)	113 (22.0)	
Female	75 (84.3)	400 (78)	
Duration (months)	96 (36‐138)	72 (24–120)	.066
Current drinking (%)	2 (2.2)	16 (3.1)	.998
Current smoker (%)	8 (9)	46 (9)	.999
Hypertension (%)	31 (34.8)	147 (28.7)	.258
Diabetes mellitus (%)	12 (13.5)	62 (12.1)	.727
WBC (×10^9^/L)	7.36 (5.74‐10.06)	6.84 (5.43–8.51)	.035
Lymphocyte (×10^9^/L)	1.75 (1.26‐2.30)	1.62 (1.25–2.12)	.334
Neutrophils (×10^9^/L)	4.53 (3.55‐7.22)	4.34 (3.25–5.77)	.099
NLR	2.96 (1.98‐4.30)	2.55 (1.89–3.75)	.116
RBC (×10^12^/L)	3.70 (3.36‐4.25)	3.99 (3.70–4.31)	.001
Hemoglobin (g/L)	103 (87.5‐116.5)	110 (98–121)	.006
TC (mmol/L)	4.33 (3.62‐5.01)	4.37 (3.71–5.09)	.695
LDL (mmol/L)	2.72 (2.19‐3.40)	2.86 (2.25–3.48)	.539
HDL (mmol/L)	1.13 (0.89‐1.40)	1.20 (0.95–1.49)	.158
TG (mmol/L)	1.11 (0.91‐1.41)	0.97 (0.74–1.32)	.003
ESR (mm/h)	55 (26.5‐76.5)	42 (22.5–63.0)	.019
CRP (mg/dL)	36.9 (15.35‐76.8)	23.3 (19.22–62.40)	.016
UA (μmol/L)	358 (269.5‐441.5)	270 (214–324.5)	<.001
Serum creatinine (μmol/L)	88 (77.5‐103)	59 (50–68)	<.001
Cystatin C (mg/L)	1.50 (1.33‐1.75)	0.97 (0.85–1.11)	<.001
RF positivity (%)	410 (79.9)	74 (83.1)	.564
Anti‐CCP seropositivity (%)	437 (85.2)	73 (82)	.428
Steroid (%)	44 (49.4)	206 (10.2)	.104
bDMARDs (%)	15 (16.9)	84 (16.4)	.878
NSAIDs (%)	88 (98.9)	484 (94.3)	.105

*Note*: values for categorical variables presented as *N* (percentage); values for continuous variables presented as mean ± *SD* or median (interquartile range, IQR).

Abbreviations: anti‐CCP, anti‐cyclic citrullinated peptide antibody; bDMARDs, biological disease‐modifying antirheumatic drugs; CRP, C reactive protein; eGFR, estimated glomerular filtration rate; ESR, erythrocyte sedimentation rate; HDL, high‐density lipoprotein; IQR, interquartile range; LDL, low‐density lipoprotein; NLR, neutrophil to lymphocyte ratio; NSAIDs, nonsteroidal anti‐inflammatory drugs; RA, rheumatoid arthritis; RBC, red blood cell; RF, rheumatoid factor; RI, renal impairment; TC, total cholesterol; TG, triglyceride; WBC, white blood cell; UA, uric acid.

### 3.2 Subgroup analysis

The results of the subgroup analysis were shown in Table 2. Our analysis indicated that female (OR = 0.523, 95% CI: 0.318–0.867, *p* = .011), neutrophils greatert than 7.5 × 10^9^/L (OR = 2.314, 95% CI: 1.310–4.087, *p* = .004), NLR > 3.53 (OR = 1.757, 95% CI: 1.104–2.799, *p* = .018), hemoglobin less than 120 g/L (OR = 2.413, 95% CI: 1.418–4.118, *p* = .001), and UA > 360 μmol/L (OR = 6.052, 95% CI: 3.708–9.878, *p* < .001) were significantly associated with renal impairment in RA patients.

**Table 2:** Subgroup analysis of RA patients according to glomerular filtration rate

**Variables**	**No. of patients**	**OR (95% CI)**	***p***
Age (years)			
<60	297	Ref	
>60	305	1.379 (876, 2.172)	.165
Sex			
Male	475	Ref	
Female	127	0.523 (0.318, 0.867)	.011
Duration (years)			
<10	375	Ref	
>10	227	1.379 (0.876, 2.172)	.165
Hypertension			
No	424	Ref	
Yes	178	1.331 (0.827, 2.142)	.241
Diabetes mellitus			
No	528	Ref	
Yes	74	1.134 (0.584, 2.201)	.711
Current drinking			
No	584	Ref	
Yes	18	0.714 (0.161, 3.161)	.657
Current smoking			
No	548	Ref	
Yes	54	1.003 (0.456, 2.203)	.995
Steroid			
No	352	Ref	
Yes	220	1.457 (0.927,2.289)	.101
bDMARDs			
No	503	Ref	
Yes	99	1.035 (0.567, 1.890)	.910
NSAIDs			
No	434	Ref	
Yes	168	5.149 (0.692, 3.827)	.110
Neutrophils (×10^9^/L)			
<7.50	470	Ref	
>7.50	132	2.314 (1.310, 4.087)	.004
NLR			
<3.53	403	Ref	
>3.53	199	1.757 (1.104, 2.799)	.018
Hemoglobin (g/L)			
>120	510	Ref	
<120	92	2.413 (1.418, 4.118)	.001
TC (mmol/L)			
<5.7	533	Ref	
>5.7	69	1.098 (0.474, 2.547)	.827
LDL (mmol/L)			
<3	373	Ref	
>3	229	1.126 (0.711, 1.783)	.612
HDL (mmol/L)			
<1.4	566	Ref	
>1.4	36	0.926 (0.349, 2.448)	.876
TG (mmol/L)			
<1.7	525	Ref	
>1.7	77	1.474 (0.797, 2.728)	.216
ESR (mm/h)			
<15	64	Ref	
>15	538	1.731 (0.909, 3.296)	.095
CRP (mg/L)			
<10	153	Ref	
>10	449	1.0444 (0.620, 1.758)	.870
UA (μmol/L)			
<360	494	Ref	
>360	108	6.052 (3.708, 9.878)	<.001

Abbreviations: bDMARDs, biological disease modifying antirheumatic drugs; CI, confience interval; CRP, C reactive protein; ESR, erythrocyte sedimentation rate; HDL, high‐density lipoprotein; LDL, low‐density lipoprotein; NASIDs, nonsteroidal anti‐inflammatory drugs; NLR, neutrophil to lymphocyte ratio; OR, odds ratio; Ref, reference; TC, total cholesterol; TG, triglyceride; UA, uric acid.

### 3.3 Multivariable logistic regression analysis

Variables (sex, neutrophils, NLR, and UA) with significant significance in subgroup analysis were incorporated in the multivariable logistic model. We employed an unadjusted and adjusted I model (covariates: age, duration, NLR, CRP, ESR, TG) to assess the independent effects of neutrophils on eGFR by multivariable logistic regression analysis. Adjusted I model analysis showed that neutrophil count greater than 7.5 × 10^9^/L (OR = 1.784, 95% CI: 1.164–3.288, *p* = .031) is an independent indicator of an increased risk of renal damage in RA patients. We also found that UA > 360 μmol/L (OR = 6.119, 95% CI: 3.708–10.099, *p* = .002), hemoglobin less than 120 g/L (OR = 2.538, 95% CI: 1.423–4.526, *p* < .001) were independently associated with a high risk of renal impairment in RA patients. The result of multivariable logistic regression analysis was presented in Table 3.

**Table 3:** Multivariate logistic regression model of RA participant with renal impairment

	**Unadjusted OR (95% CI)**	***p***	**Adjust I OR (95% CI)**	***p***
Sex				
Male	Ref		Ref	
Female	1.177 (0.615, 2.251)	.623	1.321 (0.675, 2.585)	.417
NLR				
<3.53	Ref		Ref	
>3.53	1.384 (0.800, 2.394)	.245	0.298 (0.761, 2.442)	.298
Neutrophils (×10^9^/L)				
<7.50	Ref		Ref	
>7.50	1.754 (1.033, 2.977)	.037	1.784 (1.164, 3.288)	.031
Hemoglobin (g/L)				
>120	Ref		Ref	
<120	2.412 (1.360, 4.281)	.003	2.538 (1.423, 4.526)	.002
UA (μmol/L)				
<360	Ref		Ref	
>360	5.819 (3.504, 9.665)	<.001	6.119 (3.708, 10.099)	<.001

*Note*: Adjusted I model adjusts for: age, duration, CRP, ESR, TG.

Abbreviations: CI, confidence interval; CRP, C reactive protein; ESR, erythrocyte sedimentation rate; NLR, neutrophil to lymphocyte ratio; OR, odds ratio; Ref, reference; TG, triglyceride; UA, uric acid

## 4 DISCUSSION

In this retrospective cohort study, 14.8% of patients with RA had an eGFR<60 ml/min/1.73m2, evaluated using the CKD‐EPI formula. Our data are roughly consistent with findings in previous studies. In a cohort of 400 subjects with RA in England, 13% had an eGFR < 60 ml/min/1.73 m^2^, estimated by the Modification of Diet in Renal Disease equation.^3^, ^4^ Another cross‐sectional study of 107 RA patients and 76 patients with serum‐negative arthritis observed renal impairment (eGFR < 60 ml/min/1.73 m^2^) in 17.48% of the patients with RA.^9^ A multicenter cross‐sectional survey involving 970 patients in France found that approximately 9% of patients with RA had impaired renal function, indicated by an eGFR < 60 ml/min/1.73 m^2^.^18^

In this investigation, we found that neutrophil count greater than 7.5 × 10^9^/L was associated with an increased risk of renal damage in RA patients. To our knowledge, this was the first survey to report the relationship between neutrophils and renal damage in RA patients. Previous reports suggested that oxidative stress induced by increased neutrophil count was associated with adverse renal outcomes.^19^ The property of neutrophils to release extracellular traps was not conducive to the clinical development of RA.^20^ It is well‐known that a large number of neutrophils accumulate in the synovial tissue of RA, which spontaneously released neutrophil extracellular traps (NETs).^21^ NETs, a network structure consisted of DNA and granulose, could indirectly impair the endothelial function，promote blood vessel and glomerular damage, thus it would induce renal failure and even death.^22^ Several previous studies respectively reported that the imbalance between the emergence and removal of NETs adversely affected kidney health.^23^, ^24^

Another finding of our present study showed that UA > 360 μmol/L and hemoglobin less than 120 g/L were significantly associated with a higher risk of renal impairment in RA patients. Similarly, Daoussis et al. also indicated that an elevated UA level was a strong predictor of renal damage in RA patients.^3^, ^4^ A possible explanation for this relationship may be the reduction of uric acid excretion due to renal dysfunction.^25^ Previous studies supported the fact that elevated UA levels affect kidney function by causing renal cortical vasoconstriction, intrarenal vascular disease (hypertension), and renal organ damage.^26^ Hyperuricemia played a role in renal vascular injury in healthy rats and kidney‐damaged rats, as observed in animal experiments.^27^ However, Wolfe and Michaud^28^ found that decreased hemoglobin levels were weakly associated with renal impairment. Previous studies have demonstrated that decreased hemoglobin levels were more common in patients with CKD,^29^ which may be due to shortened red blood cell lifespan and iron loss in hemodialysis patients,^30^, ^31^ erythropoietin deficiency,^32^ inflammation,^33^ iron and vitamin deficiency,^34^, ^35^ or multiple organ dysfunctions.^36^

Chiu et al followed more than 12,000 RA patients for five years in a cohort study, and they observed that diabetes, hypertension, hyperlipidemia, and cardiovascular disease were related to renal damage,^37^ which was consistent with the observations of Couderc et al.^19^ and Vansijl et al.^14^ Unfortunately, we failed to find an independent link between CV risk factors and kidney damage. This discrepancy was likely due to the smaller sample size and lack of long‐term follow‐up in our study. As was found by Haroon et al.,^9^ this study found that corticosteroids, bDMARDs, and NSAIDs usage were not independently associated with kidney damage. Previous evidence showed that the use of NSAIDs, methotrexate, tumor necrosis factor, and bDMARDs (such as etanercept) did not add to the burden of kidney function of RA patients,^38^, ^39^ but only long‐term use of cyclosporine A and cyclophosphamide caused renal damage in patients with RA.^40^, ^41^ Although there are many drugs widely used in the clinic, new extraction methods to obtain natural products may also be beneficial to the treatment of RA with renal damage.^42^

Several limitations in this study should be discussed. eGFR, estimated by various quantitative equations, has been determined to be the optimal indicator of renal function in recent years. The CKD‐EPI SCr/CysC equation recommended as a GRF equation by the KDIGO guidelines,^43^ has the advantage of more accurate calculated results, narrower calculation error, and broader clinical application.^44^, ^45^ Therefore, the CKD‐EPI equation was chosen to estimate renal function. However, the development of the CKD‐EPI SCr/CysC equation did not take into account differences in race, region, and medical facilities amongst patient cohorts, resulting in potential errors in this study's Chinese cohort in this study.^41^ Moreover, the SCr component of the CKD‐EPI equation was linked to total muscle mass, and some RA patients also present with sarcopenia. Consequently, CKD‐EPI SCr/CysC equations that depend on SCr to assess eGFR may overestimate kidney function in RA patients.^46^ Information on the disease progression of RA and the composition of specific medications (such as bDMARDs) prescribed to each patient would have improved the estimation of renal function in the cohort of this study, however, missing data disqualified any analysis regarding these two factors in this cohort. In addition, data on urinary protein levels and hematuria, both of which are markers of renal dysfunction, were not collected in the data set. Finally, the vast majority of study participants were Chinese, so the results of this study may not be generalizable to other races. Finally, our survey was a retrospective analysis, and we were unable to clarify the causality.

## 5 CONCLUSION

In this present study, neutrophils greater than 7.5 × 109/L may be an independent risk factor for renal impairment in RA. Although more prospective studies are needed to verify our results, based on the above data. we believe that continuous monitoring of neutrophils in RA patients is necessary and it could be used for early detection and identification of changes in eGFR, allowing doctors to treat patients with potential kidney damage promptly.

## CONFLICT OF INTERESTS

The authors declare that there are no conflict of interests.

## AUTHOR CONTRIBUTIONS

Pei‐Dan Yang, Si‐Min Lin, and Zhi‐Xin Chen completed collecting, analyzing the data, and drafting the manuscript. Kai‐Jun Zhu helped revise the manuscript. Min‐Ying Liu, Qing‐Ping Liu, and Chang‐Song Lin helped collect the data. Cong‐Qiu Chu and Qiang Xu conceived of the study, and participated in its design and coordination, and revised this manuscript.

## Data Availability

Data that support the findings of this study are available from the corresponding author on reasonable request
